# Individual variation in animal communication: from species averages to unique voices

**DOI:** 10.1111/brv.70061

**Published:** 2025-07-30

**Authors:** Angèle Lombrey, Marlen Fröhlich

**Affiliations:** ^1^ Paleoanthropology Section, Institute for Archaeological Sciences, Department of Geosciences University of Tübingen Rümelinstrasse 23 Tübingen 72070 Germany; ^2^ DFG Centre for Advanced Studies “Words, Bones, Genes, Tools” University of Tübingen Tübingen 72076 Germany

**Keywords:** behavioural reaction norm, communicative plasticity, cross‐species approach, individual signatures, language evolution, personality, sociodemographic factors

## Abstract

The comparative study of communicative behaviour in non‐human animals, especially primates, has yielded crucial insights into the evolution of human language. This research, mostly focused on the species and population level, has improved our understanding of the various socio‐ecological factors that shape communication systems. However, despite the inherent flexibility of human communication, the impact of individual variation on non‐human communication systems has often been overlooked, along with its potential to shed light on the roots of human language. While the eco‐evolutionary relevance of genetic and phenotypic differences between individuals is well established, animal communication studies have traditionally focused on group averages and treated outliers as noise. In this review, we address this gap by providing a comprehensive overview of the sources of individual variation in animal communicative behaviour (e.g. physiological, sociodemographic, or personality traits), across parameters such as signal forms, repertoires, and usage strategies. In particular, recent evidence from comparative work underscores the potential evolutionary significance of individual plasticity in communicative behaviour. We argue for an explicit focus on within‐individual variation and propose advancing the study of animal communication through multi‐level approaches that integrate intrinsic and environmental factors, as well as between‐ and within‐individual variation. Such approaches not only refine our view of complexity in animal communication systems and their implications for social evolution, but also help trace the evolutionary trajectory of human language through comparative studies.

## I. INTRODUCTION

Traditionally, animal communication has been understood as a comparative field: taking humans as a starting point, much ethological research focusing on animal signals has aimed to elucidate the origins of key aspects of human communication, such as intentionality, reference and syntax. Accordingly, the communication of non‐human species has mostly been studied at the species level (Bradbury & Vehrencamp, 1998), and often also from a population and cultural perspective (e.g. Henry *et al*., 2015). By contrast, individual differences have rarely been investigated explicitly in the field of animal communication. This is surprising since the consequences of between‐individual variation are well studied in a variety of other domains. For example, genotypic and phenotypic variability among individuals can impact different aspects of the ecological success of populations and species, such as their vulnerability to environmental change, their fluctuations in size, their colonisation and establishment success (Forsman & Wennersten, 2016). Between‐individual variation in behavioural expression has been shown to have important eco‐evolutionary consequences, for example, for population dynamics, life‐history trade‐offs, patterns of survival and social evolution (Wolf & Weissing, 2012; Dingemanse & Araya‐Ajoy, 2015). If individual variation can have such important consequences when present at so many levels of animal biology, there is good reason to assume that this is also the case for their communicative systems.

At the same time, complex communication systems can be inherently flexible: humans interpret and produce signals based on common ground with their communication partner (Tomasello, 2008), and several signal combinations or syntactic structures can be used to express the same message (Ferreira, 1996). In addition, combinatorial structures have been identified in Japanese tits (*Parus minor*; Suzuki, Wheatcroft & Griesser, 2016), showing similarities between human and non‐human communicative systems and raising questions about the extent of variation in non‐human communication. Based on what we know about the flexibility (also called behavioural plasticity) in the behaviour of non‐human animals, further investigation of the extent of individual‐level variation in the communicative systems of the animal kingdom would not only improve our understanding but may also help to infer the evolutionary trajectory of human language through comparative research, since other building blocks of language are already known to be shared by other species (Fitch, 2010; Van Schaik, 2016).

Moreover, behaviours are under the same selective pressure as genetic and physiological traits: natural selection is measured by the covariance between traits and fitness (Endler, 1986), and behavioural syndromes (the correlation between the average expression of behavioural traits in an individual) impose strong evolutionary constraints. Namely, without consistent individual variation, there is no opportunity for selection and hence adaptive evolution (Dingemanse & Dochtermann, 2013; Contreras Kallens, Dale & Christiansen, 2018). Such individual differences are well studied in human communication: we know, for example, that humans of the same culture can vary widely in several communicative traits (Holtgraves, 1997; Park *et al*., 2012; Aledavood *et al*., 2015), but the extent of individual variation in communicative behaviour of non‐human animals is underexplored. Yet, the similarities and differences between animal communicative behaviour and ours can help to decipher which components were inherited from common ancestors and which appeared later on in evolution (Wilke *et al*., 2017).

This review aims to provide an overview of the current state of research on individual differences in non‐human animal communicative behaviour. First, we highlight how socio‐demographic factors can impact communicative traits expressed by different individuals. Second, we stress the prevalence of individual signatures across taxa. Third, we explore whether and how personalities (“behavioural types”) are reflected in communicative signalling. Finally, we will discuss individual plasticity and the ability of individuals to shift their communicative behaviour in response to environmental changes. Together, we aim to emphasise the importance of considering all types of individual variation in the field of comparative communication and beyond, and to propose new approaches to take these studies to the next level.

## II. WHEN ARE INDIVIDUALS EXPECTED TO DIFFER IN COMMUNICATIVE BEHAVIOUR?

**Fig. 1:**
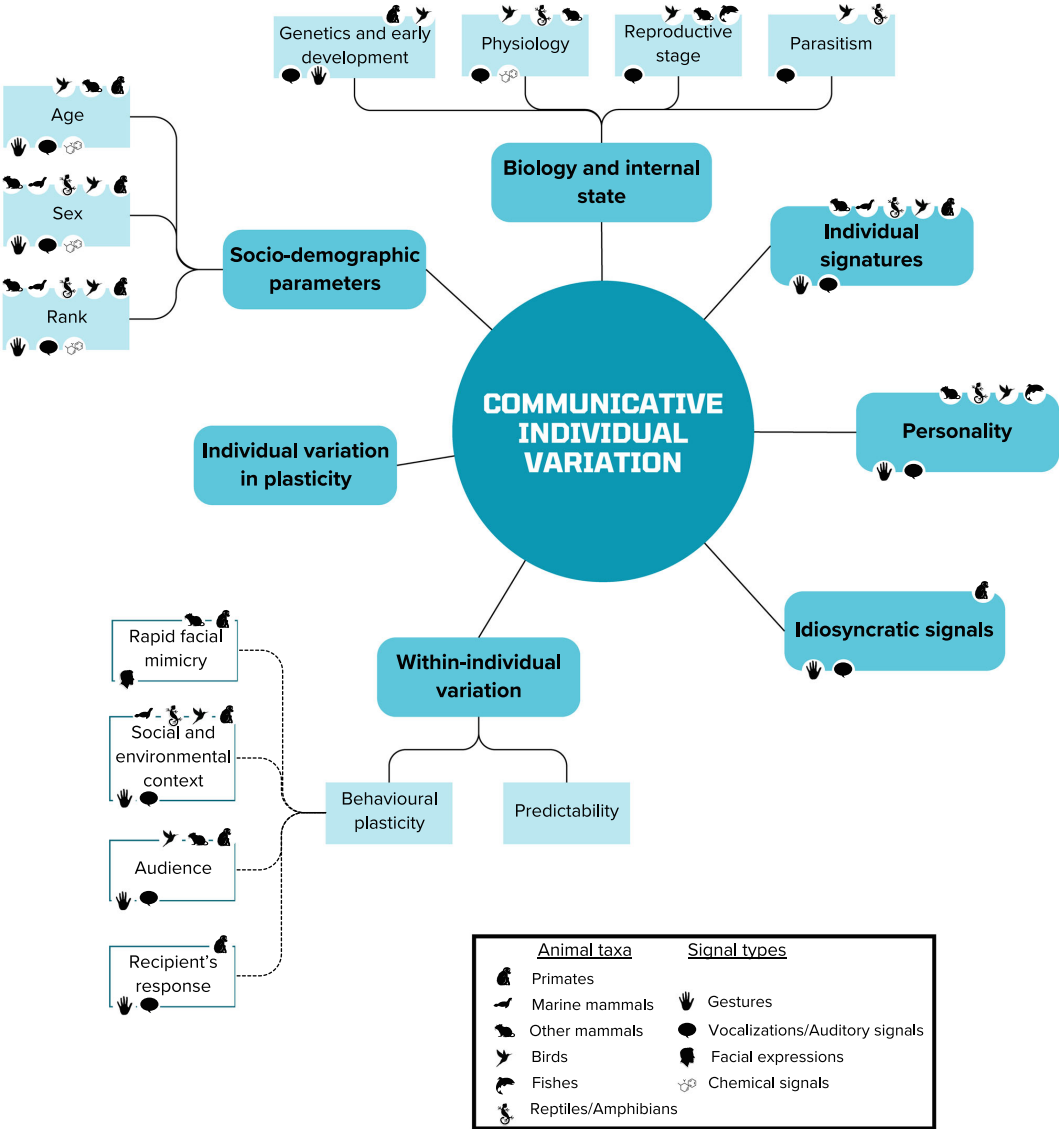
Overview of the various sources of individual variation in communicative behaviour discussed in this review. For each source and its subcategories, the animal taxa (top icons) as well as the signal types (bottom icons) in which it has been identified are presented.

Many demographic and genetic factors can vary between individuals in the same group or population, and these are known to cause behavioural differences between them: in Arctic charr (*Salvelinus alpinus*), inter‐individual variation in food intake and growth rate was greater when individuals were reared in groups than in isolation (Jobling & Baardvik, 1994); dominant vervet monkeys (*Cercopithecus aethiops sabaeus*) were shown to be more socially competent and less opportunistic than subordinate individuals, while females were more opportunistic than males (McGuire, Raleigh & Pollack, 1994). We find that communicative behaviours are affected in the same way by these factors. Animal communication is often highly complex and can involve multiple signal types, modalities and sensory channels, providing just as many opportunities for differences to arise between individuals. Although some of the most prominent causes of these differences are presented here (Fig. 1), and certain static communicative traits (e.g. linked to body size and physiology) are briefly discussed, it is beyond the scope of this review to cover them all in detail [but see Fröhlich & Hobaiter (2018) and Graham *et al*. (2022) for a focus on communication in great apes]. Our primary focus is on *dynamic signals*, specifically the variation in communicative behaviour between and within individuals. Although chemical secretions and vocal signals rely on physiological substrates (chemical composition and acoustic parameters, respectively) which may differ by sex, age, or rank, individuals can still actively control their use. They can switch these signals on or off (e.g. to advertise identity or social/physiological status) depending on context or social goal, which distinguishes them from *static signals*, such as ornaments, which are not under voluntary control. Similarly, although static individual cues like primate females' sexual swellings (Huchard *et al*., 2009) differ in shape between individuals, it is their dynamic use (e.g. “present” gestures advertising reproductive state to specific mating partners) that we are interested in here.

### (1) Genetics and early development

Given what we know about genetic variance and the heritability of behaviour within populations of the same species (Kagan, Reznick & Snidman, 1988; Magurran, 1990; Sluyter, Oortmerssen & Koolhaas, 1996; Dingemanse *et al*., 2002), we would expect genetic make‐up to have a profound influence on the variation in communicative behaviour as well. One cross‐fostering experiment with barn owls (*Tyto alba*) showed that most of the acoustic parameters expressed in the nestling calls (namely duration, amplitude, frequency and amplitude modulation) were related to the nest of origin rather than the nest of rearing. This suggests that either genetics or early development plays a major role in the ontogeny of vocal signals in this species (Dreiss, Ruppli & Roulin, 2014). The social environment in the early years of primates has also been shown to impact the ontogeny of their communicative behaviour. In chimpanzees (*Pan troglodytes*), for example, infants of more social mothers express higher gestural frequency and repertoire size (Fröhlich *et al*., 2017), possibly as a consequence of the wider range of social partners and situations to which they are exposed. Similarly, the development of facial expression processing skills of infant rhesus macaques (*Macaca mulatta*) is influenced by early social contact, mainly through maternal care (O'Callaghan *et al*., 2025). However, empirical evidence on the relationship between genetics and communicative behaviour remains scarce.

### (2) Sex

The sex of individuals affects their social behaviour in many ways, including their communicative strategies. Males and females face different challenges in navigating their social environment, which can affect both signalling repertoires and strategies. One consequence of this can be seen in chimpanzee infants, where males have a larger gestural repertoire than females (Fröhlich *et al*., 2017).

In some non‐human primates, sex differences are expressed in the duration of vocalisations or parts of vocalisations, although these differences appear to be primarily related to body size (Ey, Pfefferle & Fischer, 2007): in chacma baboons (*Papio cynocephalus ursinus*), males are larger and their wahoo calls have a longer onset than the female equivalent (Fischer *et al*., 2002), while in cotton‐top tamarins (*Saguinus oedipus*) and common marmosets (*Callithrix jacchus*), females are larger and produce longer calls (Miller, Scarl & Hauser, 2004; Pistorio, Vintch & Wang, 2006). Physical characteristics as well as sex hormones also impact vocal features in humans: the vocal fold is enlarged by increasing amounts of testosterone, resulting in the lowering of the fundamental frequency (e.g. Hollien, Green & Massey, 1994).

However, sex differences in the acoustic features of vocal communication are not necessarily related to physical characteristics. In birds, for example, the spectral energy distribution of peach‐fronted conures' (*Aratinga aurea*) contact calls differs between the sexes, with females putting more energy into higher frequencies than males, despite the lack of morphological differences between the sexes (Thomsen, Balsby & Dabelsteen, 2013). Similarly, the calls of black‐legged kittiwakes (*Rissa tridactyla*) are sexually dimorphic, with females signalling at a lower fundamental frequency and rhythm than males (Aubin *et al*., 2007).

In Mysore day geckos (*Cnemaspis mysoriensis*), male chemical secretions contain cholesterol (the major sterol present in animal fats and oils) and squalene (an organic compound precursor of sterol biosynthesis) that female secretions do not, and which is used to signal sex in intersexual communication: these components elicit a response in females but not in males, who require an additional visual signal to respond (Kabir, Radhika & Thaker, 2019; Joshi, Ellsworth & Thaker, 2022). Squalene appears to be a common sex indicator in reptile chemical communication, as it is more abundant in (although not exclusive to) the secretions of male *Trogonophis wiegmanni*, *Blanus cinereus* and garter snakes (Mason *et al*., 1989; López & Martín, 2005, 2009). In addition, female *T. wiegmanni* secrete higher proportions of steroids compared to males (Martín *et al*., 2023).

Sex differences are also expressed more broadly at the repertoire level: some signals are used specifically by one sex and not the other. This is the case in blue whales (*Balaenoptera musculus*): while D calls associated with foraging are expressed by individuals of both sexes, A and B calls have only been recorded in males and supposedly have a reproductive function (Lewis *et al*., 2018). In South American sea lions (*Otaria flavescens*), high‐pitched calls, barks, growls and exhalations were identified exclusively in males, while females expressed “mother primary calls” and grunts (Fernández‐Juricic *et al*., 1999). Male pumas (*Puma concolor*) exhibit longer communicative behaviours and higher rates of scraping and body rubbing than females, who spend more time investigating conspecific signals and express more flehmen responses and caterwauling than their male counterparts (Allen, Wittmer & Wilmers, 2014).

Differences in communication between males and females are mainly predicted by different life‐history patterns, ecological constraints and reproductive strategies. For example, male pumas rely more on signal production to find potential mates, while females tend to limit the risk of dangerous interactions with males and therefore rely more on their detection (Allen *et al*., 2014). Another explanation may be that, in species without physical sexual dimorphism, individuals rely more on differentiated communicative features to detect mating partners, and to respond appropriately to individuals of different sexes (Aubin *et al*., 2007; Thomsen *et al*., 2013).

### (3) Age

As with sex differences, age has a substantial effect on the communicative behaviour of individuals, mainly because body size tends to increase with age, which has a major impact on the vocal tract and the resulting vocalisations (Ey *et al*., 2007). Since many vocal signals are directly influenced by physical characteristics (quality, body size, etc.), signal features (e.g. fundamental frequency in vocalisations) alone can be highly informative about the individuals producing them, and recipients and/or bystanders can use this information to respond appropriately. For example, not only are the vocal parameters of barn owl nestling calls related to the caller's position in the age hierarchy, but siblings are able to modulate their own call level based on this information, in order to win a negotiation more efficiently or to avoid too heavy a loss when faced with an older sibling (Dreiss *et al*., 2014). However, gestural communication is affected by age in a way that is not related to body size. Younger individuals may need to explore their social relationships, learn context‐appropriate communicative tactics and how to use signals efficiently, so their communicative patterns may change as they gain interactional experience. Olive baboons (*Papio anubis*) not only reduce their gestural repertoire as they age, but also switch the modality of their gestures, producing fewer tactile and more audible gestures (Molesti, Meguerditchian & Bourjade, 2020). This is also the case in chimpanzees, where the use of auditory and visual gestures increases with infant age (Fröhlich, Wittig & Pika, 2016; Hobaiter, Byrne & Zuberbühler, 2017). Juveniles communicate primarily in play contexts and with their mothers, who they tend to be in close proximity to, which may favour the expression of these signal types (Hobaiter *et al*., 2017). Similarly, older spider monkeys (*Ateles geoffroyi*) use fewer tactile gestures than juveniles, but they are also more likely to consider their recipients' attentional state (Villa‐Larenas *et al*., 2024), as do other apes (Amici & Liebal, 2022). With age, an individual's communicative repertoire becomes more specific and less influenced by their emotional state: young chimpanzees, orangutans (*Pongo abelii*) and siamangs (*Symphalangus syndactylus*) use gestural sequences more frequently than adults, and not only when their social goal is not achieved (Hobaiter & Byrne, 2011a; Amici & Liebal, 2022). Adult individuals are also more interested in the communicative signals of others, such as adult pumas who investigate the olfactory signals of others significantly more than do juveniles (Allen *et al*., 2014). This is, of course, influenced by the life‐history stage that individuals are in: adults are sexually mature and need to find mates, which is not the case for younger individuals. Similarly, signals related to territory management, such as chimpanzees pant‐hoots, only become relevant later in the life of individuals as the range of challenges they face increases.

### (4) Rank

In social species, rank position can also influence communicative strategies. Dominance status is often a reflection of individual quality and fighting ability, and thus of several physiological parameters. Individuals may therefore advertise their rank through specific signalling features. For example, in the amphisbaenian (a group of reptiles characterised by reduction or loss of the limbs) *B. cinereus*, higher concentrations of squalene lead to higher levels of aggression in other males, suggesting that squalene concentrations in male secretions are an indicator of their dominance status (López & Martín, 2009). Other molecules in the femoral secretions of Iberian rock lizards (*Lacerta monticola monticola*) have the same effect (Martín, Moreira & López, 2007). In an experiment, Desjardins, Maruniak & Bronson (1973) showed that male house mice (*Mus musculus*) urinate in a pattern that depends on their social status: dominant individuals mark the entire cage floor, while subordinates mark only a few spots. Drickamer (2001) later showed that higher‐ranking males scent‐mark more than lower‐ranking males. Moreover, the vocalisations of dominant male rock hyraxes (*Procavia capensis*) have lower formant frequencies than those of subordinates (Koren & Geffen, 2009). These differences in signalling behaviour result from the need for higher ranking individuals to assert their dominant status to group members.

At the same time, lower‐ranking individuals need to invest effort in being accepted by others, maintaining affiliative relationships, and avoiding attacks from higher‐ranking individuals. Indeed, because of the risk of being detected by the dominant individuals in the flock, subordinate male red junglefowl (*Gallus gallus*) often use visual‐only signals towards females, whereas dominants always use conspicuous multimodal displays (Smith, Taylor & Evans, 2011). In chimpanzees, the pant‐grunt is a submissive vocalisation and is therefore never produced by dominant males (Goodall, 1986; Laporte & Zuberbühler, 2010). In addition, with the exception of alpha males, the proportion of gestural–vocal signal combinations produced increases with individuals' rank (Hobaiter *et al*., 2017). This rank effect has also been identified in the facial expressions of crested macaques (*Macaca nigra*), where individuals of similar rank signal with higher intensity than when the rank difference between the interaction partners is greater (Clark *et al*., 2022). The social condition associated with rank thus has a direct impact on the signalling behaviour.

### (5) Reproductive stage

As with age and sex, different reproductive stages can induce morphological variation which in turn impacts individual differences in communicative traits. Male plainfin midshipman fish (*Porichthys notatus*) use their swim bladder for acoustic communication, which changes shape depending on the reproductive stage of the individual. The enlarged bladder sonic muscle of reproductive males allows them to produce advertisement calls, which is not possible for non‐reproductive males (Rogers *et al*., 2023).

To reproduce successfully, females need to signal their readiness to copulate, so it is likely that communicative traits are affected by different phases of the reproductive cycle. Female African elephants (*Loxodonta africana*) rumble more during the anovulatory follicular phase than during other phases of the cycle, and these two types of rumble also show structural differences (Leong *et al*., 2003; Soltis, Leong & Savage, 2005). This may be explained by the greater efficiency of both males and females in detecting and signalling imminent rather than current ovulation, given the large distances between individuals in this species.

Parental experience and stage also seem to influence the way animals communicate. For instance, parenting domestic canaries (*Serinus canaria*) call less frequently than nulliparous individuals (Lalot & Bovet, 2023). In common kestrels (*Falco tinnunculus*), parents express increasing rates of alarm calls throughout the breeding season, as well as shorter syllables and lower frequencies during the incubation period compared to other stages (Huo *et al*., 2021), showing the evolution of parental investment as breeding progresses.

### (6) Internal state and health

Physiological factors can also have a physical effect on signal production, which in turn causes variation between individuals. Evidence for this effect comes mainly from the vocal domain. For example, although controversial in mammals (Fitch & Hauser, 2002; Fischer *et al*., 2004), the decrease in call frequency with increasing body size is well documented in anurans (Gingras *et al*., 2013; Tonini *et al*., 2020; Augusto‐Alves, Dena & Toledo, 2021), following the theory of acoustic allometry (Ryan, 1988; Fletcher, 2004), as is the case in European treefrogs (*Hyla arborea*) where body size affects the spectral characteristics of the mating calls (Castellano *et al*., 2002). In birds too, increased body mass can reduce song frequency: male common loons (*Gavia immer*) that gain mass over time also produce lower frequency yodels, a territorial vocalisation. As a consequence, larger individuals, which tend to have physical advantages, are more prone to sing and thus advertise their physical characteristics than smaller individuals, which have limited fighting ability and are more vulnerable to conspecific encounters (Mager, Walcott & Piper, 2007). In mammals, the songs of rock hyraxes are influenced by body size and androstenedione levels, with the former affecting lung size and thus the number of syllables that can be contained in one breath, and the latter affecting the vocal fold size and the spectral properties of songs (Koren & Geffen, 2009), while body size affects the duration of vocalisations in rhesus macaques (Hammerschmidt *et al*., 2000). In Iberian rock lizards, body size affects the chemical composition of male femoral gland secretions, which then contain higher levels of cholesterol that can be detected by conspecifics, thus influencing the outcome of social interactions (Martín & López, 2007). Similarly, steroid concentrations in amphisbaenian secretions reflect physiological parameters of individuals, such as their body size, condition and quality (Martín *et al*., 2023).

Health status can also affect vocal signals: parasitic infections negatively affect call rate and duration in frogs (Pfennig & Tinsley, 2002; Madelaire, José da Silva & Ribeiro Gomes, 2013) and great tits (Bischoff, Tschirren & Richner, 2009), reduce vocal performance (in terms of song type, rate and frequency bandwidth) in black‐striped sparrows (*Arremonops conirostris*) (Lopez‐Serna, Gonzalez‐Quevedo & Rivera‐Gutierrez, 2021), limit repertoire size in sedge warblers (*Acrocephalus schoenobaenus*) (Buchanan *et al*., 1999), and generally prevent animals from expressing more complex and challenging communicative behaviours. By contrast, calls of male Brazilian treefrogs (*Boana albomarginata*) are longer, lower pitched and produced at shorter intervals when in optimal body condition (Augusto‐Alves, Höbel & Toledo, 2024).

As mentioned above, the audience of chemical or vocal communicative signals can use the information provided by these differences to adjust their own behaviour. For example, male koalas (*Phascolarctos cinereus*) use size‐related information conveyed by the vocal features of their competitors to adjust their own vocalisations (Charlton, Whisson & Reby, 2013). This ability to detect and interpret signals is essential for conflict avoidance, where males assess each other's fighting ability prior to an actual fight, and for mate choice, where females choose males based on their quality.

## III. INDIVIDUAL SIGNATURES

Beyond these general parameters of variation, individuality in communicative behaviour can arise from intrinsic individual differences that cannot be explained by categories of individuals based on sex/age class and fitness parameters. When communicative differences are so specific that each individual has a signature, namely a stereotyped and distinctive signalling behaviour (Aubin *et al*., 2007), this enables individual recognition (Falls, 1982). Individual recognition is considered a fundamental basis for complex social interactions and a cornerstone of social evolution, as it is part of the development of individualised and stable societies (Van Schaik, 2016). Individual signatures (Fig. 1) are mostly known from vocal communication, but individual recognition can also be mediated by faeces in reptiles (Bull *et al*., 2000; Wilgers & Horne, 2009; Nisa Ramiro *et al*., 2019), and individual olfactory signatures have been identified in common marmosets (Smith, 2006). Individual signatures are much less prevalent in the visual domain (e.g. gestures and facial expressions), probably because many species rely on visual recognition of their conspecifics through physical characteristics (see Tibbetts & Dale, 2007).

Individual signatures can be widely used by social animals to discriminate between familiar and unfamiliar individuals. Group differences have been identified in hummingbird leks (Araya‐Salas *et al*., 2019), green woodhoopoe (*Phoeniculus purpureus*) (Radford, 2005) and bat groups (Knörnschild *et al*., 2012), as well as wolf packs (Zaccaroni *et al*., 2012). These group signatures are consistent over time and not driven by genetics, habitat, relatedness or sex ratio, and may facilitate territorial management and inbreeding avoidance for the dispersing sex. But even though merging into a group's signalling pattern may provide benefits, individual differences often seem to override group signatures, as in monk parakeets (*Myiopsitta monachus*) (Smith‐Vidaurre, Araya‐Salas & Wright, 2020), spotted hyenas (*Crocuta crocuta*) (Lehmann *et al*., 2022) and chimpanzees (Desai *et al*., 2022).

For signalling features to be defined as signatures, they must have high between‐individual variation, so that different individuals can be distinguished by their signals, but also be consistent, that is have low within‐individual variation. Such characteristics have been identified in the vocalisations of a wide range of species, from amphibians [green frogs (*Rana clamitans*); Bee *et al*., 2001] to birds and mammals [chimpanzees (Marler & Hobbett, 1975; Desai *et al*., 2022); southern elephant seals (*Mirounga leonina*) (Sanvito & Galimberti, 2000); leopard seals (*Hydrurga leptonyx*) (Rogers & Cato, 2002); African elephants (Soltis *et al*., 2005)], and are often used for mother–offspring recognition and cohesion, as in sea otters (*Enhydra lutris nereis*) (McShane *et al*., 1995), South American sea lions (Fernández‐Juricic *et al*., 1999) or South American fur seals (*Arctocephalus australis*) (Phillips & Stirling, 2000). But signatures are also used in more specific ways. Animals of many species possess signalling features that are used for individual recognition throughout their lives. This is the case for black‐legged kittiwakes, which are able to recognise their mates from their vocal signatures year after year (Wooller, 1978). Common dolphin (*Tursiops truncatus*) signature whistles contain identity information that is based on vocal learning and the other whistles present in the individual's environment at the beginning of its life (Janik, Sayigh & Wells, 2006; Janik & Sayigh, 2013). To ensure recognition and reduce the risk of confusion between individuals, calls may contain redundant and multi‐parametric features whose production, processing and memorisation are more cognitively demanding than passive vocal cues. This way, individual recognition remains possible even when some vocal features change due to changes in individual morphology or when the habitat provides a lot of background noise (Naguib, Hammerschmidt & Wirth, 2001; Aubin *et al*., 2007; Thomsen *et al*., 2013; Elie & Theunissen, 2018).

Beyond individual recognition, signature calls may aid in group coordination, as in dolphins, which live in a vast three‐dimensional habitat with few landmarks and thus require additional strategies to maintain contact between individuals (Janik *et al*., 2006). Similarly, in birds, vocal signatures are mostly embedded in contact calls, which can reach long distances and compensate for the lack of visibility (Elie & Theunissen, 2018) or serve as a reminder of past social interactions when choosing who to associate with (Thomsen *et al*., 2013). Contact calls are also a cheaper way than singing to advertise territory ownership (Naguib *et al*., 2001). In barn owl and Jackson's golden‐backed weaver (*Ploceus jacksoni*) nestlings, individuality is particularly detectable when hungry, probably because it gives them a competitive advantage by deterring siblings from joining a contest for food (Reers & Jacot, 2011; Dreiss *et al*., 2014). In dolphins, individuality is mostly expressed in one specific signature whistle, which in turn has a specific function, but in zebra finches (*Taeniopygia guttata*) it is present, at least to some extent, in all call types, making individuals recognisable at all times and across contexts (Elie & Theunissen, 2018).

Although they have been most extensively studied in the vocal domain, individual signatures are not restricted to vocalisations. Chimpanzee drumming bouts during travel events also encode individual signatures, in contrast to those produced during dominance displays (Eleuteri *et al*., 2022). Both pant‐hoots [which also carry individual signatures (Marler & Hobbett, 1975; Desai *et al*., 2022)] and drums seem to serve primarily as long‐distance signals for mediating fission–fusion dynamics, which is part of the group coordination function mentioned above: chimpanzees combine the two signals in travel contexts, where advertising one's identity may be advantageous to promote subgroup fusion and avoid unwanted social interactions (Eleuteri *et al*., 2022). However, little is known about consistent individual differences in primate communicative interactions at close range.

In sum, individual signatures allow mutual recognition of conspecifics and group members and, as such, must be distinct for each individual and consistent over time. Accordingly, there can be great individual variation in a variety of communicative features, from compositional (e.g. frequency, chemical composition) to behavioural (e.g. rhythmicity) parameters.

## IV. IDIOSYNCRATIC SIGNAL USE

Differences between the communicative characteristics of individuals may not only come from species‐specific traits. In all great ape species, individuals have been observed to express gestures that were not displayed by other individuals [chimpanzees (Tomasello *et al*., 1985, 1994; Goodall, 1986); bonobos (*Pan paniscus*) (Pika, Liebal & Tomasello, 2005; Halina, Rossano & Tomasello, 2013); gorillas (*Gorilla gorilla*) (Pika, Liebal & Tomasello, 2003); orangutans (Liebal, Pika & Tomasello, 2006)], and idiosyncratic vocalisations may have recently been identified in orangutans (Roth *et al*., 2024), as well as an idiosyncratic hand clap gesture in a Barbary macaque (*Macaca sylvanus*; Bosshard *et al*., 2024). These so‐called idiosyncratic signals (i.e. signals that are unique to an individual (Tomasello *et al*., 1994) (Fig. 1) may arise in individuals that are particularly motivated to achieve a certain goal (Halina *et al*., 2013), and are another source of variation in communicative repertoire between individuals. The occasional production of such signals argues for individual learning processes rather than genetic hard‐wiring. As mentioned above, individual differences are the basis for evolutionary processes to take place: idiosyncratic signals have the power to shape the evolution of communicative traits through ontogenetic ritualisation, which would be one of the processes of gestural acquisition, by combining repeated social interaction and individual learning processes (Tomasello & Zuberbühler, 2002; Pika *et al*., 2003; Fröhlich & Hobaiter, 2018). Although it is often unclear whether idiosyncratic signals truly indicate an individual process or are merely artefacts of insufficient sampling, they contribute to communicative differences between individuals and may thus impact social dynamics.

## V. PERSONALITY EXPRESSED IN THE INDIVIDUALISATION OF COMMUNICATIVE BEHAVIOUR

While individual signatures are generally embedded in the structure of signals, such as acoustic features or chemical composition, and idiosyncratic signals may arise sporadically in an individual's repertoire, individuals may also differ consistently across time and contexts in more general communicative strategies. This variation may reflect differences in personality (Fig. 1; Dingemanse & Réale, 2005). Communication mediates social interactions, and the transmission of personality information through communicative signals could shape the nature of social relationships between individuals (Friel *et al*., 2016). Indeed, we already know that friendships in chimpanzees are based on personality similarities between individuals (Massen & Koski, 2014), who may therefore need to recognise the personality types of their conspecifics easily in order to choose with whom to associate.

Recognition of personality by other individuals may also be an advantage in mate choice: individuals who are more inclined to take risks are more attractive to females and may have higher mating success than their less‐bold counterparts, as bold behaviour may reflect higher male quality. Such a relationship has been observed in collared flycatchers (*Ficedula albicollis*), where males singing on a lower and more exposed tree post had higher mating success than individuals singing higher in the canopy (Garamszegi, Eens & Török, 2008). This type of personality information can be conveyed *via* communicative signals, as in male great tits, where a relationship between pre‐breeding explorative behaviour and singing activity has been found (Naguib *et al*., 2010). Moreover, as song composition is highly dependent on the vocal environment, more exploratory individuals may encounter a wider range of songs, including rarer features, that shy individuals may not, resulting in differences in repertoire and song parameters (Garamszegi *et al*., 2008).

The expression of personality through communicative signals has been explored by relating consistent communicative patterns expressed by individuals to a well‐known set of personality parameters, such as boldness. For example, there is a tendency for song duration to be related to exploration in collared flycatchers (Garamszegi *et al*., 2008). In pigs (*Sus scrofa domestica*), the rate of acoustic signalling is strongly related to personality, as proactive individuals vocalise at a higher rate than reactive ones and are therefore more likely to be detected by group members. As proactivity is associated with aggressiveness in this species, obtaining this information from conspecifics' vocalisations could help to assess their fighting ability (Friel *et al*., 2016).

Personality‐related variation in communicative behaviour can also be expressed in conflictual contexts. Great tits that were faster in exploration tests changed song type significantly more often when faced with a simulated territory intrusion, and vocalised more in the presence of a predator, compared to slower individuals that instead approached the threat more often (Hollander *et al*., 2008; Jacobs *et al*., 2014). However, other studies in this species have reported conflicting results (Amy *et al*., 2010), likely due to the different playback methods used (Jacobs *et al*., 2014), demonstrating that these types of studies are highly method sensitive. Nonetheless, a similar correlation was found in the vocal response to stressful contexts in black‐capped chickadees (*Poecile atricapillus*; Guillette & Sturdy, 2011), but not in superb fairywrens (*Malurus cyaneus*) which showed no exploratory score‐dependent differences in their response to territory intrusion (Hall *et al*., 2017). In song sparrows (*Melospiza melodia*), aggressiveness consistently predicted vocal and visual signalling levels (Akçay, Campbell & Beecher, 2014) and the likelihood of producing alarm calls in response to playback of conspecific alarm calls (Hyman, Myers & Krippel, 2013).

A relationship between personality types and visual communication was even identified in Siamese fighting fish (*Betta splendens*). Individuals that were persistent in their aggressive signalling expressed a higher duration and frequency of gill cover display compared to more unpredictable individuals (Matessi *et al*., 2010). In contrast, no difference was observed in their tail beating frequency, demonstrating that not all communicative variables are necessarily influenced in the same way by personality types.

To date, very little research has been carried out on the relationship between personality and communication in primates. However, in a study based on zookeeper ratings, extravert chimpanzees were found to engage in more affiliative behaviour with group members, and personality traits were generally a predictor of individuals' social behaviour (Pederson, King & Landau, 2005). Since social behaviour necessarily involves communication, it is reasonable to presume that personality may have an impact on the apes' signalling behaviour, although this remains to be tested.

A key reason why personality and communication might be linked is that many communicative signals, and vocalisations in particular, are influenced by the emotional state of the signaller, which in turn is linked to their stress response (Friel *et al*., 2016). Communicative patterns could thus be regulated by the neuroendocrine system, which has already been associated with consistent behavioural differences between individuals (Koolhaas *et al*., 2010), sympathetic nervous system reactivity or corticosterone response (Cockrem, 2007).

## VI. WITHIN‐INDIVIDUAL VARIATION ACROSS CONTEXTS AND AUDIENCES

So far, we have discussed consistent differences in communicative behaviour between individuals, whether due to differences in internal biology, signatures or personality (Fig. 1). But an individual might not always express his communicative behaviour in a consistent, fixed manner across time: this is the expression of within‐individual variation. Within‐individual variation partly reflects the extent to which individuals consistently respond in the same way to the same conditions, that is their predictability (Hertel *et al*., 2020; O'Dea, Noble & Nakagawa, 2022). Namely, individuals might express more or less variability around their behavioural type (i.e. their average behavioural expression), which is a reflection of their specialisation (Hertel *et al*., 2020). As very little research has been carried out on predictability in communicative behaviour, this section will focus on the second aspect of within‐individual variation: an individual's phenotype can be significantly altered by its environment, either in an irreversible way (developmental plasticity) or in a reversible way for phenotypes that are not determined by past conditions (Dingemanse & Wolf, 2013). Thus, an individual can flexibly adjust its behaviour to changes in its immediate environment: this is the expression of its behavioural plasticity (Fig. 1). Individuals can benefit from this when their environment is variable and they need to express the adaptive behavioural response (Dall, Houston & McNamara, 2004; Hall *et al*., 2017). In communicative interactions, the risks that individuals lacking plasticity may face range from failure to achieve their social goal to aggressive responses from conspecifics. These so‐called “environmental changes” can be as diverse as the behavioural adjustments they entail.

### (1) Environmental and social context

#### (a) Change of signal parameters between contexts

The context (i.e. the circumstances or situation in which the interaction takes place) can strongly affect the signalling behaviour of individuals. This information can be reflected in the structure of communicative signals, such as their acoustic features, which can be modified as required by the immediate context. In common kestrels, the number of offspring affects the acoustic parameters of the parental alarm calls, suggesting that parents invest more energy in nest defence when the number of offspring is higher (Huo *et al*., 2021). The acoustic parameters of baboons' wahoo calls differ between contexts: “wa” syllables have a lower frequency and “hoo” syllables are louder and longer in competitive contexts compared to alarm contexts (Fischer *et al*., 2002). Similarly, the pant‐hoot of chimpanzees (a species‐specific long‐distance call) has more let‐down and build‐up exhalation elements and fewer climax elements in travel contexts than in feeding contexts (Desai *et al*., 2022), and macaques use facial expressions at a higher intensity when involved in high‐risk interactions (e.g. aggression, copulation) than in affiliative interactions (Clark *et al*., 2022). Such contextual adjustments in the structure of communicative signals (vocal, facial or gestural) are common in primate species (e.g. van Hooff, 1973; Liebal *et al*., 2014). Changes in the social environment, whether it is long or short term, also have an impact on communicative features. Pygmy marmosets (*Cebuella pygmaea*), for instance, change the acoustic features of their contact “trill” calls when placed in the vicinity of another population in order to make the calls of both populations more similar (Elowson & Snowdon, 1994; Snowdon & Elowson, 1999). Vocal matching to the interaction partner has also been found in female Diana monkeys (*Cercopithecus diana*) and male chimpanzees (Mitani & Brandt, 1994; Candiotti, Zuberbühler & Lemasson, 2012).

For acoustic communication to be effective, however, recipients must be able to hear the signals targeted at them. Yet, the perception of acoustic signals can easily be impaired by the sound environment. Thus, many species modify the amplitude of their vocalisations according to ambient noise levels. In general, they increase their sound level as the ambient noise increases, a phenomenon known as the Lombard effect. This has been observed in a variety of species, including birds (Manabe, Sadr & Dooling, 1998; Cynx *et al*., 1998; Kobayasi & Okanoya, 2003), mammals, but also humans (Lane & Tranel, 1971; Amazi & Garber, 1982). Domestic chickens (Brumm, Schmidt & Schrader, 2009) and common marmosets (Brumm *et al*., 2004) increase the amplitude of their calls with increasing levels of background noise, demonstrating that they can assess their environment and modulate their vocal production accordingly. This behavioural adjustment maintains an optimal signal‐to‐noise ratio to avoid signal masking and ensure successful communication.

However, not all species use the Lombard effect to adjust to environmental noise. Harbour seal pups (*Phoca vitulina*) shift their communicative behaviour in the opposite direction, lowering the fundamental frequency of their vocalisations as noise levels increase, revealing a specialisation in low‐frequency signals (Torres Borda *et al*., 2021). If the Lombard effect can allow individuals to rise above ambient noise, this contrasting strategy seems to focus on better airborne propagation of lower frequencies to reach greater distances.

Vocal flexibility is even evident in reptiles. Tokay geckos (*Gekko gecko*) do not vary the intensity of specific call types, but rather choose to express higher‐amplitude syllables from their vocal repertoire more than lower‐amplitude syllables (Brumm & Zollinger, 2017). Geckos are thus able to control their vocalisations to maximise their communicative success. In addition, tokay geckos as well as common marmosets can improve signal detection by increasing the duration of their calls, as longer sounds are more easily detected by the recipient's auditory system (Brumm *et al*., 2004; Brumm & Zollinger, 2017). This may be an adaptation to the limited visibility in forest environments: since they cannot rely on visual cues to signal their position effectively to their groupmates, individuals need to make their vocal signals efficient in a varying acoustic environment, and one way to do this is to take into account the perception system of their targets. Marmosets also change the frequency of their phee calls depending on the frequency of ambient noise: Zhao, Rad & Wang (2019) showed that the frequency of these calls increased in a high‐frequency noise condition, but decreased in a low‐frequency noise condition. Furthermore, these findings were consistent when subjects were switched from one experimental set‐up to another, demonstrating that individuals can voluntarily control the structure of their vocalisations and specifically adjust them to the immediate sound context in which they find themselves.

#### (b) Change of communication strategies between contexts

Individuals can also shift their communicative strategies under different social or physical conditions. Namely, they can adapt the type of signals they use and the way they use them. For example, Jacky dragons (*Amphibolurus muricatus*) increase the proportion of tail flicks in their displays with increasing wind speed as an adjustment to the increasing visual noise (i.e. colourful or agitated environment, due to constant wind), which presumably interferes with the detection of their bodily gestures (Ramos & Peters, 2017). Wild spider monkeys are more likely to use tactile and visual gestures when the subgroup engages in social contexts than in foraging or travelling contexts (Villa‐Larenas *et al*., 2024) and orangutan mothers may persist in their interaction with their infants, or respond to their infant requests at different rates depending on the context they are in (Fröhlich *et al*., 2022), while rhesus macaque and gorilla mothers use exaggerated communicative displays with young infants that they do not use with adults (Ferrari *et al*., 2009; Luef & Liebal, 2012).

### (2) Audience

In terms of communication, contextual changes can be linked not only to the individual's socioecological environment, but also specifically to their audience. Many species have been shown to adjust their signalling to the attentional state of the recipient. For instance, gorillas specifically use visual gestures when the recipient is attentive, whereas there is no difference for tactile gestures (Pika *et al*., 2003). Similar results have been found for the other great ape species (Tomasello, 2008; Dafreville *et al*., 2021; Amici & Liebal, 2022) and also for olive baboons (Molesti *et al*., 2020), generally in favour of the use of tactile signals when the recipient is not looking. Simultaneously, young orangutans have been shown to express play face with varying levels of both intensity and complexity depending on the attention state of their recipient: their open mouth facial expression was more intense and involved more facial action units when the recipient's view was unobstructed (Waller, Caeiro & Davila‐Ross, 2015). Primates are thus able to select the signal types that are most likely to lead to successful communication based on the attentional state of the intended recipient, but they are not alone. Elephants use their visual and auditory repertoire much more than their tactile repertoire when the recipient is visually attentive. When the recipient is not attentive, their use of tactile signals increases at the expense of silent visual signals (Eleuteri *et al*., 2024). Subordinate male chickens also adjust their multimodal signalling to their audience's attentional states; interestingly, not to that of females, but to that of rival males: they use unimodal, silent displays when the dominant male is attentive, instead of implementing calls in a multimodal display as they do when there is no risk of eavesdropping (Smith *et al*., 2011). In this case, signalling modulation is driven by the cost of being noticed.

Competition for food is another situation in which individuals may adjust their signalling in response to the individuals around them. In barn owls, for example, nestlings produce more, louder, and longer food calls, but also lower‐pitched calls, in the presence of older siblings. As call frequency decreases with age in this species, call modification may be a way to outcompete older and more competitive siblings and increase the likelihood of being fed (Dreiss *et al*., 2014).

In some species, the specific social bond between the interaction partners also impacts the communicative strategies they might use. Carolina chickadees (*Poecile carolinensis*) tend to call more during flight and earlier after the presentation of a threat when in presence of familiar than unfamiliar flockmates (Coppinger, Sanchez de Launay & Freeberg, 2018). Wild chimpanzees switch the modality of their gestural signalling based on the relationship with their recipient: visual gestures are preferred within dyads having stronger social bonds, while loosely bonded dyad rely more on auditory and tactile gestures (Roberts & Roberts, 2016), and young chimpanzees prefer to use tactile gestures in their food and play solicitations with their mothers and visual ones with other individuals (Fröhlich *et al*., 2016, 2020). Furthermore, the degree of familiarity with their human recipient influences the strategy used by bonobos in case of communicative failure: signal repetition is used more with familiar recipients, while signal elaboration (i.e. the use of different signals) is preferred with unfamiliar recipients (Genty, Neumann & Zuberbühler, 2015).

### (3) Response

Plasticity can also be seen in the response to communicative signals. Campbell's monkeys (*Cercopithecus campbelli*) show changes in their vocal variants over time, and groupmates are able to discriminate between them: playback of a female's former variants does not elicit a response from other group members, whereas current variants do, showing that Campbell's monkeys keep up with vocal changes in their conspecifics (Lemasson, Hausberger & Zuberbühler, 2005). Similarly, persistence and specifically elaboration (i.e. modification of signal use) of signalling reflects plasticity in reaction to a lack of understanding by the interaction partner or failure to achieve one's goal. Orangutans can discriminate between different levels of understanding on the part of their interlocutor and to adjust their subsequent signalling accordingly: they use different signals when their initial message is not understood, whereas they repeat their previous signals when their interlocutor has partially understood them (Cartmill & Byrne, 2007). Similar results were found in chimpanzees, who stopped signalling when their goal was achieved, elaborated (i.e. continued to gesture with multiple signals) when their goal was partially achieved, and elaborated even more (i.e. used entirely different signals) when their communicative attempt failed (Leavens, Russell & Hopkins, 2005).

### (4) Rapid facial mimicry

Finally, rapid facial mimicry (RFM) can be considered a form of behavioural plasticity, as it involves the ability to copy the emotional expressions of others, even when they do not match one's own (Davila‐Ross & Palagi, 2022). Facial mimicry is crucial in human social interactions (Chartrand & van Baaren, 2009) and has also been studied in all great ape species (Davila Ross, Menzler & Zimmermann, 2007; Davila‐Ross *et al*., 2011; Bresciani, Cordoni & Palagi, 2022), in geladas (*Theropithecus gelada*) (Mancini, Ferrari & Palagi, 2013), and in meerkats (*Suricata suricatta*) (Palagi *et al*., 2019). Moreover, this reaction is often impacted by the social relationship with the interaction partner (Davila Ross *et al*., 2007; Bresciani *et al*., 2022), emphasising the plasticity of this behaviour. Given the risk of aggressive responses to misinterpreted playful signals, matching facial expressions and laughter is a way to share and maintain playful intentions (Davila‐Ross *et al*., 2011).

All of the types of plasticity presented here can only be observed by looking at variation at the individual level, that is, consistent variation irrespective of the other factors discussed in this review. To date, although recent studies have begun to investigate communicative flexibility more thoroughly (Fröhlich *et al*., 2022; Amici & Liebal, 2024), studies of contextual variation and audience effects have mostly been conducted at the population or group level, and behavioural variation across contexts has rarely been studied within the same individuals, highlighting an important area for future investigation. This is particularly true when considering how primates excel in this area, which is a key prerequisite for complex communication and human language, as it is this ability that allows the combination of repertoire elements, grammar and syntax to emerge.

## VII. IMPLICATIONS AND FUTURE RESEARCH AVENUES

The evidence for biologically meaningful individual differences in non‐human communicative behaviour is overwhelming, and the sources of those differences are multi‐faceted (Fig. 1). As emphasised above, individual variation is an essential element for adaptive evolution (Dingemanse & Dochtermann, 2013; Contreras Kallens *et al*., 2018). Between‐individual differences in behavioural type, namely the average expression of a chosen behaviour, define the type of habitats or social environments in which animals can live (Holtmann *et al*., 2017), and differences in behavioural plasticity reflect their different ability to adjust to rapidly changing environments. More plastic individuals may thus be able to cope with a wider range of situations, while less‐flexible individuals may need to remain in a relatively stable environment, which in the long term and through the fitness consequences of such variation may shape species evolution (Ghalambor *et al*., 2007; Edelaar, Jovani & Gomez‐Mestre, 2017). This is likely also to be the case in communicative behaviour, where more plastic individuals may be able to form a greater number and more stable relationships with group members, or be more successful in their communicative attempts. Thus, individual differences in communicative traits are a key element for understanding the properties and evolution of complex communication systems across the world.

As a consequence, studying the effects of between‐ and within‐individual variation in the communicative behaviour of non‐human species can provide key insights into the evolutionary trajectory of human language, which exhibits a degree of plasticity unparalleled in the animal kingdom. Human communication is exceptionally tailored to the social context, the interaction partner (“audience design”; Bell, 1984) and their attentional state, as well as to shared interaction histories (Tomasello, 2008). Some of these features have also been identified in other species (see above) and especially in the great apes [e.g. in gorillas (Poss *et al*., 2006); orang‐utans (Liebal *et al*., 2006); chimpanzees (Hobaiter & Byrne, 2011b); bonobos (Genty *et al*., 2014)], showing that some key features of language are not unique to humans, and raising questions about the processes involved in making human communication so peculiar.

For species‐wide communication to be effective, individuals must share a repertoire that can be understood by conspecifics. Communication thus includes a set of innate signals and behaviours (e.g. Genty *et al*., 2009; Byrne *et al*., 2017) that limit the range of variation between individuals. These signals are further refined through social learning and shaping in several species (e.g. Janik & Slater, 2000). Together, these processes create a trade‐off between individual and group/species‐level variation (Fröhlich, Boeckx & Tennie, 2025), where individuals must strike a balance between individuality and species conformity. However, despite this necessary common ground, it is well established that individual animals can vary greatly in their average level of communicative behaviour across different contexts. This variation can be driven by factors such as sociodemographics, health, or “personality”. In the past decade, a growing number of studies have begun to consider that individuals also differ in their responsiveness to environmental variation (i.e. plasticity). Although the two phenomena have largely been studied separately, we now know that within‐ and between‐individual variation can be correlated. Recent studies on several animal species (e.g. Fairbanks, 1996; Torres Borda *et al*., 2021) have indeed revealed between‐individual variation in the extent of behavioural flexibility, due to combined effects of genetics and environment (Dingemanse & Wolf, 2013). Dingemanse *et al*. (2010) argue that it is essential to analyse these two complementary aspects together in order to grasp their function and gain the most comprehensive insight into behavioural variability.

Behavioural reaction norm (BRN) frameworks allow this interaction to be examined by plotting between‐individual variation in plasticity and personality along an environmental gradient: the intercept reflects the individual's average behaviour (their behavioural type) while the slope reflects behavioural plasticity (if the slope is zero, the individual does not express context‐driven variation in their behavioural response), providing two parameters around which individual BRNs can vary independently (Dingemanse *et al*., 2010; Dingemanse & Wolf, 2013; Hertel *et al*., 2020; Fröhlich *et al*., 2022). Such a method has already been applied to social and spatial behaviour in several species (Montiglio *et al*., 2010; Carter, Goldizen & Heinsohn, 2012; Hertel *et al*., 2020), but virtually nothing is known about the extent of within‐ relative to between‐individual variation in communicative behaviour (Fröhlich *et al*., 2022).

Adopting the BRN approach is new to the field of communicative behaviour, but has recently been successfully applied to examine infant‐directed communication in orangutan mothers (Fröhlich *et al*., 2022). The authors used generalised linear mixed models (GLMMs) with a fixed effect of context to assess plasticity (i.e. whether individuals adjusted their behaviour across different contexts), and included a random intercept for individual ID and a random slope for context within individual ID, allowing the slope (i.e. the change in behaviour across contexts) to vary between individuals. This approach was used to examine individual differences in both behavioural type and plasticity. Importantly, it also allows the simultaneous analysis of variation at both the species and group levels, as the effect of each variable is estimated in relation to the others. However, it requires a substantial data set; ideally, a large number of repeated observations from a large number of individuals. The mothers of two different species and research settings showed significant differences in the modification of their behaviour across social goals, with some expressing a greater responsiveness to contextual changes than others, implying that the degree of behavioural plasticity varies among individuals. These results show that mother–infant communicative behaviour is highly variable among individuals, but also highly plastic. This method allowed successful identification of both population‐ and individual‐level variation. Considering how rarely comparative research on non‐human species' communication takes an individual‐centred approach, our review aims to emphasise how much remains to be learned.

## VIII. CONCLUSIONS

(1) The purpose of this review was to summarise current knowledge about the sources of individual variation in communicative behaviour. Not surprisingly, these sources were found to be multi‐faceted, ranging from physiological (e.g. body size, health) and socio‐demographical (e.g. sex, rank) parameters to intrinsic, consistent sources (i.e. signatures, personality). All of these need to be considered when studying communicative behaviour since they have major consequences, whether on a few features of a communicative signal or the entire behavioural repertoire. Failure to account for these confounding variables can lead to misinterpretation of communicative behaviour, with results that may depend on a few specific individuals.
(2) Another facet of individual variation is the extent to which individuals can change their behaviour in response to their immediate environment (i.e. individual plasticity). Communicative behaviour is usually studied at the population or species level, where main results equate to mean values for a communicative response variable. However, individuals of multiple species have the ability to adjust to a long list of circumstances: environmental and social context, audience, recipient response, and conspecific emotional state. The study of within‐individual variation is a necessary next step in better understanding non‐human communication.
(3) To take the integration of individual variation into the study of communicative behaviour even further, an interesting future avenue would be to investigate the extent of between‐ and within‐individual variation collectively. The behavioural reaction norm framework, derived from studies in behavioural ecology, would be an ideal way to do this, since it allows exploration of whether individuals adjust to changing environments in the same way, or whether, on the contrary, they follow distinct patterns. Promising results have already been obtained in primates, but there is a severe lack of data in this area.
(4) Quantifying the extent of individual variation in behaviour is critical not only for understanding animal communication systems, but also the evolution of human communication. Individual variation is one of the materials on which natural selection and evolution are based, which makes it a key concept in deciphering the evolutionary trajectory of language, and this can only be achieved through a comparative approach.
